# Editorial: The interaction between digestive tract microbes and hosts in poultry

**DOI:** 10.3389/fvets.2022.972769

**Published:** 2022-09-27

**Authors:** Yan Hu, Shourong Shi

**Affiliations:** ^1^Poultry Institute, Chinese Academy of Agriculture Science, Yangzhou, China; ^2^Jiangsu Co-innovation Center for Prevention and Control of Important Animal Infectious Diseases and Zoonoses, Yangzhou, China

**Keywords:** poultry, digestive tract microbes, microbial metabolites and derivatives, composition and development pattern, host metabolism, target regulation

As a high-quality source of animal protein, poultry meat and egg products are an important component of a rich, healthy, and well-balanced diet for humans. In domestic animals, the health and function of the gut barrier is related to their growth and performance. The intestinal microbiota and their products are an important part of the intestinal mucosal barrier, and they have a profound influence on the functioning of the intestine and thus of the host. Studies in mammals have shown that the intestinal microbiota is essential for gut development, maturation, metabolism, and immune programming. In birds, the intestinal microbiota also plays an important role in host physiology (1). However, the characteristics of the colonization and prevalence of intestinal microbiota in poultry have not been well studied. A better understanding of the assembly and community composition of the microbiota in discrete periods of life will therefore help elucidate the important role of the microbiome in poultry physiology (1). Previous studies have confirmed that the structure of the intestinal microbiota (including its diversity and abundance) is highly plastic and modifiable, and that the age of the host has a dramatic effect on the colonization and prevalence of various microbes, as well as on some of their unique regulatory functions. The effect of the host's growth and aging on the colonization and prevalence of digestive tract microbiota, particularly on microbial function and metabolites, still needs further study in poultry.

Unlike mammals, birds have a relatively short growth cycle, but the composition and proportions of the dominant intestinal microbiota during the early-life period are quite different from those during the adult-life period (1). In Arbor Acres (AA) broiler chickens, *Firmicutes* (F) (90.50%), *Proteobacteria* (5.57%), and *Tenericutes* (2.99%) made up 99% of the cecal microbiota during the first phase of life (days 1–21). The proportions of these three phyla in the cecum were reduced to 28.52, 2.51, and 1.20%, respectively, during the second phase (days 22–42), while *Bacteroidetes* (B) was greatly increased, from 0.44 to 67.35%. The F/B ratio decreased from a value in the hundreds at 21 days of age to a few tenths at 42 days of age (i.e., by 600 times), suggesting that the F/B ratio cannot be used as a measure of gut health in birds as it is in mammals.

In this special e-collection, a long-term observation of the changes of the intestinal microbiota in hens (Sun et al.) verified that the diversity, composition, and function of the chicken intestinal microbiota were distinct in different growth periods. The dynamic succession of microbiota across the duodenum, cecum, and feces of broilers at 1, 7, 21, and 35 days of age (Zhou et al.) sheds direct light on the spatial and temporal heterogeneity of the colonization of intestinal microbiota. Moreover, several genera identified as distinct intestinal segment biomarkers also changed with the growth of the hen and across different intestinal segments. The community diversity of the cecum increased rapidly over time and gradually reached a relatively stable state, which confirms that the poultry cecum is a species reservoir of intestinal microbes.

Ma J. et al. offer a gene catalog detailing the cecal bacteria of Japanese quail, along with a discussion of gender-based differences in bacterial composition. Insights like these can foster greater understanding of the microbiota in the quail ceca. However, due to the diversity of species, genera, breeds, and lines in poultry, and due to variation in the farming scales, growing stages, environments, and/or disease models ranging from cultured communities, longitudinal observations on and research into digestive tract microbes in poultry are still lacking (2). Comparisons among different breeds or lines are even scarcer.

Over the past decade, combined omics-informed studies have revealed that intestinal microbiota and their metabolites aid in the programming of important bodily systems such as the immune and the central nervous system during critical temporal windows of development, with possible structural and functional implications throughout the lifespan (3). A recent review of human early-life gut microbiota alteration (Guo et al.) integrated current evidence to describe the feasibility of the “able-to-be-regulated microbiota,” summarized the underlying mechanisms of the “microbiota-driven immune system education,” explored the optimal intervention time window, and discussed the potential of designing early-probiotic treatment as a new prevention strategy for inflammatory bowel disease. In particular, nutritional interventions and environmental stimulation during the early-life period are essential for the plasticity and transitivity of characteristic functional intestinal microbiota and the consequent programming of digestive tract maturation, metabolism, and immunity. This early-life period (i.e., from birth until the microbiota reach an adult or pediocratic phenotype) opens up an exciting window of opportunity.

A chick embryo model was used to investigate the effects of L-arginine *in ovo* on the intestinal development and microbial succession of embryos (Dai et al.). Modifications to the microbial assembly pattern and succession, particularly the enrichment of several short-chain fatty acid (SCFA)–producing bacteria, could mediate L-arginine supplementation, thereby improving embryonic intestine development.

In geese, persistent purine abnormalities may lead to aggravation of visceral inflammation and intestinal *Bacteroides* dysbiosis (Ma W. et al.). In broiler chickens, heat stress affected the modification of primary bile acids (BA) by altering the cecal microbiota composition (Yin et al.), leading to disturbance of BA metabolism, which probably influenced their heat stress resistance capability. Treatment of postnatal ducks with blue light (Xia et al.) significantly increased the beta diversity of their intestinal microbiota and the relative abundances of BA hydrolase–producing bacteria, which was accompanied by an increase in the leg muscle and in the relative length of the intestine. These findings may contribute to exploring nutritional strategies for establishing health-promoting microbiota by manipulating host-microbe interactions during the early-life period for the healthy development of birds.

A series of metabolites and derivatives of intestinal microbiota, such as SCFAs, lipopolysaccharides, secondary BAs, trimethylamine, imidazolpropionic acid, branch chain amino acids, and indole, can be used as messengers to influence a range of factors, including host energy homeostasis, obesity, appetite, blood sugar regulation, insulin sensitivity, inflammation, and endocrine regulation, and to regulate host metabolism (4). SCFAs are metabolites, mainly composed of acetate, propionate, and butyrate, generated by bacterial fermentation of dietary fiber in the hindgut (Liu et al.). They are primarily absorbed from the intestine and used by enterocytes as a key substrate for energy production. One crucial property of intestinal microbiota in poultry is to establish resistance against pathogen invasion and colonization by fermenting non-digestible carbohydrates into SCFAs and then releasing H^+^ and decreasing the *p*H of the hindgut. SCFAs (mainly butyrate) consume luminal oxygen to create an anaerobic environment, thereby reducing aerobic pathogens such as *Salmonella* in the gut lumen. Studies have demonstrated that SCFAs (e.g., propionate, butyrate, formate, caproate) can inhibit colonization by several pathogens, such as *Salmonella* spp., *Salmonella* typ., *Escherichia coli*, and *Shigella* spp., to maintain a stable microbial environment in the intestines of broiler chickens.

In this special e-collection, there are 10 papers which have shown that SCFAs play a significant role in the regulation of intestinal health in poultry. Several genera that are related to the production of SCFAs, including *Blautia, Ruminiclostridium* and *Ruminococcus*, were identified as biomarker bacteria of the cecum in broilers after 21 days of age (Zhou et al.) or broilers treated by L-arginine (Dai et al.), which confirms that the poultry cecum is associated with the increased prevalence of SCFA-producing bacteria.

There are also 2 papers devoted to the regulatory role of BA. It has been indicated that BA and microbiota could play an important role in gastrointestinal health. BA are produced in the liver as primary BA and metabolized in the intestine to secondary BA, which participates in modifying the microbiota (i.e., deconjugation and dehydroxylation). *Alistipes* and *Lactobacillus*, which are related to the production of secondary BA, have been identified as biomarkers in birds under heat stress (Yin et al.) or treated by blue light (Xia et al.). At present, studies on bacterial metabolites in poultry, such as trimethylamine, imidazolpropionic acid, branch chain amino acids, and indole, are relatively scarce. Increasing the understanding of the intestinal microbiota and its metabolites would be beneficial for improving the production performance and health of poultry.

Recently, there has been increasing emphasis on the importance of microbiome science in the field of human medicine, especially the intestinal microbiome, which is increasingly being recognized as an organ in its own right or even as a “second brain”. Their role in the physiological function and metabolism of other organs and tissues has been the subject of a great deal of research (3, 5). Accompanied by the specific process of intestinal microbiome colonization, the microbiota-intestinal-brain axis or the microbiota-intestinal-liver axis established in an individual during this period can potentially represent the main determinants of the life-long metabolic pattern. These axes therefore sit at the epicenter of the microbe-host interactions and the holistic health of the host. At the same time, microbial postgenomic medicine should be embraced by the animal science community, especially for the study of poultry, as it is sure to play a transformative role over the next decade.

There are two papers that have shown that some bile acids of microbial origin, such as taurine, tauro-conjugated ursodeoxycholic acid (TUDCA), and hyocholic acids (HCAs), may play important roles in maintaining homeostasis in the intestinal-liver axis by reducing lipid levels in serum and liver. This has been observed in birds under heat stress (Yin et al.) or treated with blue light (Xia et al.). In a fecal microbiota transplantation model, the intestinal microbiota community of Arbor Acres broilers was remodeled by oral gavage of a bacterial suspension derived from Beijing-You broilers (Lei et al.), suggesting that the alteration of intestinal microbiota induced certain changes in drip loss by regulating muscle fiber diameter. The population of *Lachnoclostridium* spp. was associated with drip-losing rate, meat fiber diameter, body weight, and abdominal fat rate, and was therefore identified as an indicator of meat quality. This provides theoretical evidence for the existence of the microbiota-muscle axis and a new strategy for optimizing meat quality. However, it is worth noting that in-depth studies of the interaction of molecular and physiological mechanisms between digestive tract ecosystem communities and host organs in poultry are relatively less advanced compared with those in humans, pigs, and even ruminants. Studies in poultry have, for example, focused almost exclusively on microbiota in the posterior digestive tract and have only used *in vivo* models.

Studies on the functions of intestinal microbiota have brought good news to the post-antibiotic era. New feed additives and feeding management techniques targeting intestinal microbiota and their metabolites or derivatives have received maximum attention, research, and application. In intensive modern breeding systems, domestic poultry are exposed to various challenges, such as diet changes, pathological environments, and especially the prohibition of antibiotic growth promoters (AGP), which may cause decreases in production performance, increases in incidence rates, and even death. Preliminary research on safe and effective additives that have regulating functions or are alternatives to AGP is urgently needed. Such advances could improve birds' intestinal health and maintain optimum growth, thus guaranteeing healthy farming (1).

Many studies in this collection were conducted to identify functional feed additives with similar beneficial effects as antibiotic growth promoters. They have examined a variety of ingredients, including prebiotics, probiotics, organic acids, enzymes, vitamins, essential oils, flavonoids, plant tannin, lignocellulose, and medium-chain fatty acids. The functions of these ingredients have been evaluated from the perspectives of growth performance, antioxidant enzyme activity, inflammatory cytokine concentration, and intestinal morphology, along with sieving and separating the specific strains, developing the recommended dosage and feeding management. A recent review (Zhu et al.) integrated recent discoveries on gut health maintenance through the use of these functional feed additives as alternatives to antibiotics in the past 10 years. Ultimately, the authors concluded that identifying a single “ideal” solution within the wealth of options for gut health control would be difficult.

Several measures and alternatives to antibiotics can be used in conjunction with one another to achieve optimal intestinal health. The common mechanism involved in these interventions, especially in the context of poultry health, is inseparable from the interaction between digestive tract microbes and hosts. This latter topic is in need of further extensive research.

In summary, the results of the above-mentioned studies and reviews represent new relevant data on the composition and development pattern of microbial communities in the poultry digestive tract, on the relationship between digestive tract microbiota and host physiological metabolism in poultry, and on new feed additives and feeding management technologies that target digestive tract microbiota and metabolites. Despite all the existing literature and evidence related to this extremely important topic, the papers published in this e-book clearly show that there are still many aspects to be clarified and understood in the fascinating world of interactions between digestive tract microbes and hosts, including in poultry. We hope that this editorial, along with other work on comparative longitudinal observation and research on the structure and dynamics of the microbial ecosystem in the digestive tracts of various breeds or lines, along with research on the messenger function of microbes and theirs metabolites and derivatives, will give the reader a clearer picture of the research conducted to date and reinforce the important role of the microbiome in host physiology.

## Author contributions

SS wrote the introduction and the conclusion. YH wrote the central part with comments on the cited papers and references. Both authors contributed to the article and approved the submitted version.

## Funding

This work was supported by the earmarked fund for Jiangsu Agricultural Industry Technology System (JATS[2022]406) and Su Xi Broiler Industry Cluster Project.

## Conflict of interest

The authors declare that the research was conducted in the absence of any commercial or financial relationships that could be construed as a potential conflict of interest.

## Publisher's note

All claims expressed in this article are solely those of the authors and do not necessarily represent those of their affiliated organizations, or those of the publisher, the editors and the reviewers. Any product that may be evaluated in this article, or claim that may be made by its manufacturer, is not guaranteed or endorsed by the publisher.

## References

[1] Hu Y Wang LD Shao D Wang Q Wu YY Han YM. Selectived and reshaped early dominant microbial community in the cecum with similar proportions and better homogenization and species diversity due to organic acids as AGP alternatives mediate their effects on broilers growth. Fron in Micro. (2020) 10:2948. 10.3389/fmicb.2019.02948 31993028 PMC6971172

[2] Schokker D Veninga G Vastenhouw SA Bossers A de Bree FM Kaal-Lansbergen LM. Early life microbial colonization of the gut and intestinal development differ between genetically divergent broiler lines. BMC Genomics. (2015) 16:418. 10.1186/s12864-015-1646-6 26017153 PMC4446945

[3] Cong X Henderson WA Graf J McGrath JM. Early life experience and gut microbiome: the brain-gut-microbiota signaling system. Adv Neonatal Care. (2015) 15:314–23. 10.1097/ANC.0000000000000191 26240939 PMC4583334

[4] Reid G Abrahamsson T Bailey M Bindels LB Bubnov R Ganguli K. How do probiotics and prebiotics function at distant sites? Benef Microbes. (2017) 8:521–33. 10.3920/BM2016.0222 28726511

[5] Malan-Muller S Valles-Colomer M Raes J Lowry CA Seedat S Hemmings SMJ. The gut microbiome and mental health: implications for anxiety- and trauma-related disorders. OMICS. (2018) 22:90–107. 10.1089/omi.2017.0077 28767318

